# Greenhouse and field evaluation of a novel HPPD-inhibiting herbicide, QYM201, for weed control in wheat

**DOI:** 10.1038/s41598-018-38221-y

**Published:** 2019-02-07

**Authors:** Fengwen Zhang, Shuang Bai, Hengzhi Wang, Weitang Liu, Jinxin Wang

**Affiliations:** Key Laboratory of Pesticide Toxicology and Application Technique, College of Plant Protection, Shandong Agricultural University, Tai’an, 271018 China

## Abstract

QYM201, 1-(2-chloro-3-(3-cyclopropyl-5-hydroxy-1-methyl-1H-pyrazole-4-carbonyl)-6-(trifluoromethyl)phenyl)piperidin-2-one), is a newly developed HPPD- (4-hydroxyphenylpyruvate dioxygenase; EC 1.13.11.27) inhibiting herbicide for weed control. Experiments were carried out to determine the effect of QYM201 on weeds and its safety for wheat in the glasshouse and field. The results indicated that at doses of 90 and 135 g active ingredient (a.i.) ha^−1^ QYM201 was highly effective against both grass and broadleaf weeds, such as *Alopecurus aequalis* Sobol., *Alopecurus japonicus* Steud, and *Capsella bursa-pastoris* Medic. In a wheat hybrid tolerance experiment, QYM201 showed a high level of safety for most of the 17 tested wheat hybrids, and the SI values reached ≥5.7 in the selectivity index study. To determine application rules for QYM201, field experiments were conducted in 2016 and 2017. During this time, 90 to 270 g a.i. ha^−1^ post-emergence herbicide application (POST) was sufficient to supply satisfactory all-season control of *Alopecurus aequalis* Sobol., *Descurainia sophia* [L.] Schur., and *Malachium aquaticum* (L.) Fires. No damage to wheat plants was observed. In order to increase wheat yield and deliver effective weed control, a dosage of 90 to 180 g a.i. ha^−1^ is suggested. In conclusion, the herbicide QYM201 is safe to use in wheat fields to control winter weeds.

## Introduction

Winter wheat (*Triticum aestivum* L.) is the second most widely grown food crop in China, with a planting area of 24.1 million hectares and a production of 130.2 million tons in 2015^1^. Severe wheat yield losses can be caused by weeds with potential reductions up to 15%^2,3^. *Alopecurus japonicus* Steud., *Beckmannia syzigachne* [Steud.] Fern., *Alopecurus aequalis* Sobol., *Vicia sativa* L, *Capsella bursa-pastoris* Medic., *Descurainia sophia* [L.] Schur., and *Avena fatua* L are examples of common troublesome weeds in winter wheat fields^4^. Substantial yield reduction takes place when these weeds are not fully controlled.

Herbicides have been used for weed control in China since the early 1990s^5^; nowadays, chemical weed control still has an important role in producing high-yielding crops^6^. Herbicides with different modes of action can kill 90% to 99% of target weeds and are the most useful means of weed control developed^7–9^; however, this is not problem free. One case study reveals that more than 252 weed species have developed resistance to 23 different herbicides worldwide^10^. In China, almost 30 weed species have developed resistance to nearly 50 herbicides with more than 10 different sites of action so far^11^. Most recently, Zhu *et al*. reported that at least 12 weed species have been confirmed resistant to the main herbicides commonly used in wheat fields^11^. Specifically, in Jiangsu and Anhui provinces of China, *A. japonicus*, a widespread troublesome weed, has developed resistance to about 20 herbicides. These 20 herbicides include not only Acetyl-CoA carboxylase (ACCase) herbicides such as fenoxaprop-P-ethyl, pinoxaden, clodinafop-propargyl, and sethoxydim, but also include Acetolactate synthase (ALS) herbicides: pyribenzoxim, imazapic, imsulfuron, sulfosulfuron, penoxsulam, and pyroxsulam^12^. Another problem is that some herbicides have a narrow weed spectrum and occasionally can cause damage to wheat plants; for example, Fluroxypyr is effective in controlling some broadleaf weeds but is ineffective against *C. bursa-pastoris* (L.) Medic. Mesosulfuron plus iodosulfuron can kill most weed species but sometimes damage wheat plants. Hence, the widespread use of Mesosulfuron and iodosulfuron has been limited in China. In another example, 2, 4-D butyl ester has become a commonly used herbicide in wheat fields for controlling broadleaf weeds in China, but it can cause damage to broadleaf crops due to spray drift and volatilization; this is particularly relevant with cotton^13,14^. In addition to the problems described above, extensive use of herbicides can also lead to an accelerated succession of weed communities. Therefore, herbicides with a new site of action, that have broad-spectrum weed control, high efficacy, and are safe to use on wheat are urgently needed.

**Figure 1:**
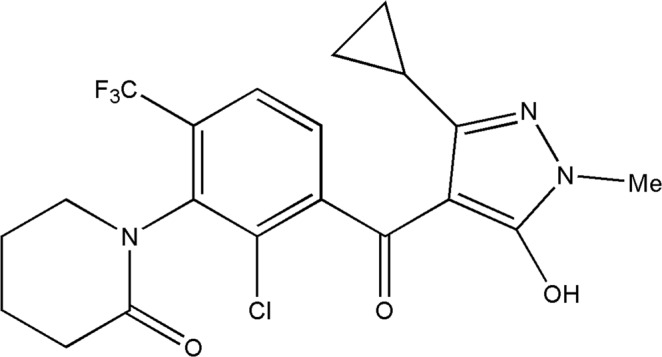
Chemical structure of the herbicide QYM201 used in this study.

QYM201, (C_20_H_19_ClF_3_N_3_O_3_, 1-(2-chloro-3-(3-cyclopropyl-5-hydroxy-1-methyl-1H-pyrazole-4-carbonyl)-6-(trifluoromethyl)phenyl)piperidin-2-one; Fig. 1), is a novel HPPD-inhibiting herbicide that was developed by Qingdao Kingagroot Chemicals Co., Ltd. in 2011^15^. 4-hydroxyphenylpyruvate dioxygenase (HPPD) is a class of α-keto acid-dependent non-heme iron (II) oxygenases which can be found in mammals, plants, and most microbes. HPPD catalyzes oxygenation of 4-hydroxyphenylpyruvate (HPP) to generate homogentisate (HG)^16–19^. The biosynthesis of prenylquinone and tocopherols is prevented once HPPD is inhibited in plants, which leads to a decrease in carotenoid biosynthesis, blocking of photosynthetic electron transfer chains, and photooxidation of chloroplasts^20,21^. Consequently, treated plants become bleached to death^21^. Therefore HPPD is selected as a target for herbicide development. To our knowledge, HPPD inhibitors have not been used in wheat fields anywhere in the world. Therefore, we suggest that QYM201 is a potentially beneficial herbicide for weed control, especially for resistant and harmful weeds in wheat fields.

In order to determine the spectrum of weed control, the safety to different wheat hybrids, and the selectivity of QYM201 among 3 commonly planted wheat hybrids and 4 common weeds, experiments were carried out in the greenhouse. In addition, field experiments were conducted to determine the effect of QYM201 on weed control in winter wheat fields with different rates of 6% QYM201 oil dispersion (OD) during the 2015–2016 and 2016–2017 growing seasons in Shandong province.

## Results

### Greenhouse experiments

#### Effectiveness of weed control

At all rates of application, QYM201 was effective on many of the tested weed species including grass weeds and broadleaf weeds. Treated weeds exhibited symptoms of bleach injury at 5 days after treatment (DAT), eventually undergoing necrosis and death at 20 DAT. At the dosage of 90 g a.i. ha^−1^, QYM201 was highly effective against 3 of the treated weeds, and dry weight inhibition of *A. aequalis, M. aquaticum*, and *D. sophia*. were up to 93%, 91%, and 92%, respectively. Meanwhile dry weight inhibition of *A. japonicus, and L. arvense* were 86% and 87%, respectively. Weed injury increased according to application rate - higher rates leading to greater injury. At a dosage of 135 g a.i. ha^−1^, 8 of the treated weeds were controlled by QYM201, with dry weight reductions ranging from 91% to 95% these weeds included *A. aequalis, A. japonicus, C. bursa-pastoris, M. aquaticum, V. didyma, P. kengiana, L. arvense*, and *D. sophia* (Table 1). However, even at high doses some weed species, such as *L. multiflorum*, *B. japonicus*, *A. squarrosa*, and *E. helioscopia*, showed only slight sensitivity to QYM201 with dry weight reductions of 21%, 23%, 20%, and 19%, respectively.

**Table 1:** Dry weight inhibitions of trial weeds treated with QYM201 relative to the non-treated control in a greenhouse study 28 days after treatment (DAT).

Trial weeds	Dry weight inhibition (SE)^a^ g a.i. ha^−1^		F-statistic	P-value
	90	135		
	%			
*Alopecurus aequalis* Sobol. (Water foxtail)	93 (0.4)	94 (0.3) NS	2.63	0.247
*Alopecurus japonicus* Steud. (Japanese foxtail)	86 (0.2)	91 (0.5)**	485.04	0.002
*Capsella bursa-pastoris* (L.) Medic (Shepherd’s purse)	85 (0.1)	91 (0.3)**	462.36	0.002
*Malachium aquaticum* (L.) Fires (Crickweed)	91 (0.1)	94 (0.4)*	45.14	0.021
*Veronica didyma* Tenore (Speedwell)	86 (0.3)	91 (0.4)*	34.26	0.028
*Becmannia syzigachne* (Steud.) Fern. (American slough grass)	80 (0.2)	83 (0.0)**	181.21	0.006
*Pseudosclerochloa kengiana* (Ohwi) (Hardgrass)	85 (0.1)	91 (0.6)*	46.13	0.021
*Poa annua* L. (Annual bluegrass)	12 (0.7)	27 (0.7)*	60.32	0.016
*Lithospermum arvense* L. (Corn gromwell)	87 (0.3)	93 (0.4)*	80.11	0.012
*Descurainia sophia (L.) Schur*. (Flixweed)	92 (0.0)	95 (0.2)**	646.02	0.002
*Geranium sibiricum* L. (Carolina cranesbill herb)	45 (0.6)	52 (0.8) NS	10.19	0.086
*Galium aparine* L. var. *tenerum* (Gren. et Godr.) Rcbb. (Catchweed)	27 (0.2)	36 (0.6)*	52.92	0.018
*Lolium multiflorum* Lam. (Italian ryegrass)	13 (0.1)	21 (1.0)*	30.74	0.031
*Bromus japonicus* Thunb. (Japanese brome)	19 (0.2)	23 (0.7)*	28.96	0.033
*Aegilops squarrosa* L. (Triticum tauschii)	14 (0.1)	20 (1.0)*	26.49	0.036
*Avena fatua* L. (Wild oat)	47 (0.7)	61 (1.0)**	531.37	0.002
*Vicia sativa* L. (Vetch)	44 (0.7)	53 (1.2) NS	6.74	0.122
*Euphorbia helioscopia* L. (Sun spurge)	14 (1.0)	19 (1.4)**	160.45	0.006

^a^Significant differences between the 2 rates at the 0.05 level according to Fisher’s protected LSD test. *Significant at P < 0.05; **significant at P < 0.01; ***significant at P < 0.001; NS, not significant.

#### Wheat hybrid tolerance

QYM201 was safe for most of the treated wheat in the greenhouse experiment. When treated at 360 g a.i. ha^−1^, most of the tested wheat hybrids were tolerant to QYM201 with reductions of <7% and herbicide damage <20% (Table 2). However, Huamai 5, Yangfumai 4, and Yangmai 158 were sensitive to QYM201, showing dry weight reductions of 13%, 12%, and 17%, respectively. The damage caused by QYM201 to these 3 hybrids ranging from 12% to 17% (Table 2). Wheat hybrids Zhengmai 10, Hengguan 35, Haomai 1, and Xinong 979 became sensitive to QYM201 with dry weights inhibited by beyond 10%, while crop injury caused by the herbicide was up to 28% at a dose of 540 g a.i. ha^−1^ (Table 2). Meanwhile, there were obvious differences in sensitivity to QYM201 among the wheat hybrids. Most hybrids showed little if any reaction to the herbicide; Shannong 22, Jinan 17, Shannong 19, Zhengmai 9023, Yannong 19, Ningmai 24, Jimai 22, Liangxing 66, and Tainong 18 exhibited no obvious damage, and reductions in plant dry weights were <10%, with herbicide damage <20%. At the beginning of the treatment, some wheat plants showed some symptoms of whitening at 5 DAT, but subsequently all of them regained a normal appearance at approximately 12 DAT (Table 2).

**Table 2:** Dry weight inhibitions (%) and visual injury ratings (%) of trial wheat hybrids treated with QYM201 as a POST relative to the non-treated control in a greenhouse study 28 days after treatment (DAT).

Wheat hybrid	Dry weight inhibition (SE)^a^		F-statistic	P-value	Wheat injury rating(SE)^a,b^		F-statistic	P-value
	g a.i. ha^-1^				g a.i. ha^-1^			
	360	540			360	540		
	%				%			
Shannong 22	1 (0.5)	3 (1.9) NS	2.34	0.266	0	0 NS	ND^c^	ND
Zhenmai 10	7 (0.0)	11 (1.8) NS	4.84	0.159	12 (1.8)	42 (0.6)**	106.21	0.009
Jinan 17	2 (0.2)	9 (0.5)**	168.83	0.006	4 (0.8)	23 (0.9)***	3172.33	0.000
Huamai 5	13 (0.1)	18 (1.8) NS	5.31	0.148	34 (2.4)	48 (0.9) NS	6.85	0.120
Shannong 19	1 (0.5)	4 (1.2) NS	10.00	0.087	0	0 NS	ND	ND
Jimai 17	2 (1.5)	10 (1.2) NS	15.41	0.059	5 (0.2)	17 (1.1)*	83.15	0.012
Yangfumai 4	12 (0.3)	14 (0.1)**	98.61	0.010	34 (1.7)	45 (0.8)*	18.65	0.050
Hengguan 35	3 (1.0)	12 (1.0)*	26.12	0.036	10 (0.9)	28 (1.2)**	1542.29	0.001
Zhengmai 9023	7 (0.1)	9 (0.9) NS	2.29	0.270	7 (1.0)	18 (0.7)**	406.75	0.003
Yannong 19	2 (0.4)	5 (0.6) NS	12.71	0.071	0	0 NS	ND	ND
Haomai 1	5 (0.7)	16 (0.1)**	321.92	0.003	10 (0.2)	32 (1.0)**	366.57	0.003
Ningmai 24	0 (1.2)	5 (1.7)**	382.07	0.003	2 (1.1)	5 (0.8) NS	4.99	0.155
Yangmai 158	17 (0.2)	21 (0.9)*	21.29	0.044	35 (1.4)	53 (0.5)*	29.50	0.032
Xinong 979	5 (0.8)	13 (0.8)*	23.52	0.040	16 (0.7)	32 (0.6)*	67.92	0.014
Jimai 22	1 (1.8)	4 (1.6)***	713.52	0.001	8 (0.6)	25 (0.9)*	87.35	0.011
Liangxing 66	2 (0.2)	8 (0.4)**	179.89	0.006	12 (0.7)	27 (0.5)**	233.06	0.004
Tainong 18	3 (0.4)	6 (0.7) NS	12.76	0.070	3 (0.8)	14 (0.8)***	3768.20	0.000

^a^Significant differences between the 2 rates according to Fisher’s protected LSD test. *Significant at P < 0.05; **significant at P < 0.01; ***significant at P < 0.001; NS, not significant. ^b^Injury rating scale: 0% = no injury, 0~30% = cotyledon and a few functional leaves showed bleaching in addition to newly-emerged leaves, 30~60% = cotyledon, minority of functional leaves and newly-emerged leaves presented bleaching, 60~100% = most plants showed sever whitening symptoms and some even showed necrosis, 100% = plant death. ^c^ND, not determined.

#### Selectivity index (SI)

**Figure 2:**
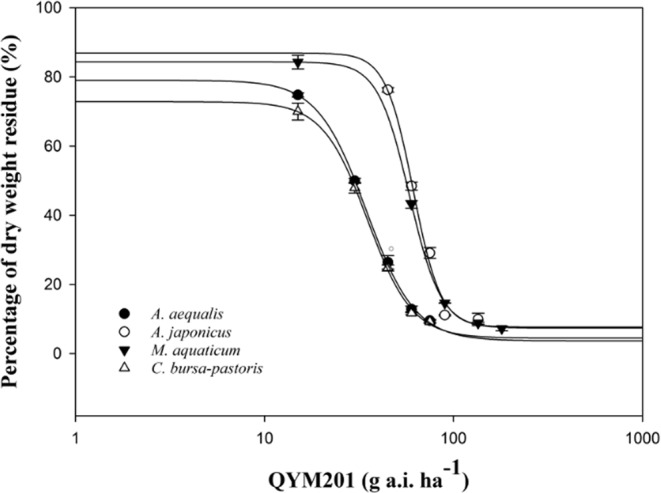
Percentage of dry weight residue of *A. aequalis*, *A. japonicus*, *M. aquaticum*, and *C. bursa-pastoris* as influenced by different doses of QYM201 at 28 days after treatment (DAT) in a greenhouse study. The regression lines were calculated using Equation 1.

In view of the wheat hybrid tolerance results, a dose-response study was performed to determine the SI values between 3 wheat hybrids (JM 22, LX 66, TN 18) and 4 weed species (*A. aequalis, A. japonicus, C. bursa-pastoris*, and *M. aquaticum*). GR_50_ values of the 3 wheat hybrids were 4856.7, 2469.2, and 6114 while the values of the 4 weed species were 28.1, 58.9, 40.5, and 24.9, respectively (Table 3). The high GR_50_ values clearly indicated that QYM201 was safe for the 3 tested wheat hybrids and that the 4 weed species were also effectively controlled (Table 3). In addition, experimental results showed that *A. aequalis* and *M. aquaticum* were more sensitive to QYM201 under post emergence herbicide applications (POST) than *A. japonicus* and *C. bursa-pastoris* (Table 3, Fig. 2). SI values from Table 3 indicate that QYM201 was safe for JM 22, LX 66, and TN 18 against weeds tested in this study with values ranging from 5.7 to 16.6.

**Table 3:** The doses of QYM201 causing 10% and 50% reduction of wheat dry weight and 50% and 90% of weeds’ dry matter, and the selectivity index (SI) values between 3 wheat hybrids and the 4 weed species 28 days after treatment (DAT) in greenhouse research.

Trial plants	GR value (SE)^a^			SI^b^		
	GR_10_	GR_50_	GR_90_	JM 22	LX 66	TN 18
	g a.i. ha^-1^					
Jimai 22	930.4 (18.9)	4856.7 (38.9)	ND^c^	ND	ND	ND
Liangxing 66	784.4 (12.8)	2469.2 (26.8)	ND	ND	ND	ND
Tainong 18	1190.6 (21.6)	6114.0 (41.6)	ND	ND	ND	ND
*Alopecurus aequalis*	ND	28.1 (3.2)	80.8 (1.9)	11.5	9.7	14.7
*Alopecurus japonicus*	ND	58.9 (2.7)	115.0 (2.6)	8.1	6.8	10.4
*Capsella bursa-pastoris*	ND	40.5 (0.8)	137.5 (3.2)	6.8	5.7	8.7
*Malachium aquaticum*	ND^c^	24.9 (1.4)	71.5 (1.1)	13.0	11.0	16.6

^a^GR, inhibitory concentration. ^b^SI, selectivity index values were calculated by equation 2. ^c^ND, not determined.

### Field experiment

Throughout the 2 years of field experiments, QYM201 performed with good efficacy against *A. aequalis, D. sophia*, and *M. aquaticum* (Table 4). The 3 weed species began turning white and stopped growing within 15 days after treatment (DAT). When treated at doses of 90–270 g a.i. ha^−1^, all weed densities decreased; moreover, control over *A. aequalis, D. sophia*, and *M. aquaticum* to levels of 90.6% to 100% was achieved in 45 DAT. Just as speculated, fenoxapro-P-ethyl had almost no effect on *D. sophia* and *M. aquaticum*, and tribenuron-methyl did not affect *A. aequalis*. QYM201 provided biomass reductions of 100% in 2016 and 99.6% in 2017 for *A. aequalis* at a dosage of 270 g a.i. ha^−1^. It supplied a high biomass reduction (>90%) even at the lower rate of 90 g a.i. ha^−1^. Furthermore, no obvious damage to the wheat crop was observed at all tested rates during 2016 and 2017 (Table 5). The grain yield of wheat increased with increasing rates of QYM201 from 90 to 270 g a.i. ha^−1^ (Table 5). Specifically, in the field experiments of 2017, a 19% yield increase was achieved by increasing the rate from 0 to 270 g a.i. ha^−1^. The yield in hand-weeded plots could be reduced by about 20% in two years, and none of the herbicide treatments led to superior grain yields compared with hand-weeded plots (Table 5).

**Table 4:** Visual estimates of percentage control of weeds under different POST rates of QYM201 at Tai’an, Shandong, China, in 2016 and 2017.

Treatments	Dose	Percent weed control^a,b^					
		*Alopecurus aequalis*		*Descurainia sophia*		*Malachium aquaticum*	
		2016	2017	2016	2017	2016	2017
	g a.i. ha^−1^	%					
QYM201	90	91.7 c	90.6 d	93.5 d	91.9 d	94.2 b	94.6 b
QYM201	135	94.7 b	92.2 c	95.8 c	95.9 c	94.6 b	95.0 b
QYM201	180	99.4 a	96.3 b	97.9 b	96.6 b	97.3 a	97.6 a
QYM201	270	100.0 a	99.6 a	99.9 a	98.5 a	98.9 a	97.8 a
Fenoxapro-P-ethyl	50	88.3 d	83.3 e	—	—	—	—
Tribenuron-methyl	22.5	—	—	76.1 e	80.7 e	91.8 c	91.6 c
Hand-weeding	—	—	—	—	—	—	—
Weedy control	—	—	—	—	—	—	—

^a^Visual estimates for weed control were recorded after 45 days of treatment, using a 0% (no weed control) to 100% (complete weed control) scale. ^b^The following different letters represent different significance at the P < 0.05 level according to Fisher’s protected LSD test.

**Table 5:** Visual estimates for wheat injury and wheat yields under different POST rates of QYM201 at Tai’an, Shandong, China, in 2016 and 2017.

Treatments	Dose	Crop injury^a,b^								Wheat yield^b,c^		Yield growth rate^c^	
		3 DAT		5 DAT		15 DAT		30 DAT					
		2016	2017	2016	2017	2016	2017	2016	2017	2016	2017	2016	2017
	g a.i. ha^−1^	%								kg ha^−1^		%	
QYM201	90	0	0	0	0	0	0	0	0	6136 (34)^b^	5930 (103)^d^	9.8	11.8
QYM201	135	0	0	0	0	0	0	0	0	6321 (270)^ab^	6099 (32)^bcd^	13.1	15.0
QYM201	180	0	0	0	0	0	0	0	0	6411 (246)^ab^	6231 (23)^bcd^	14.7	17.5
QYM201	270	0	0	0	0	0	0	0	0	6529 (229)^ab^	631 1(46)^ab^	16.8	19.0
Fenoxapro-P-ethyl	50	0	0	0	0	0	0	0	0	6009 (132)^bc^	5885 (109)^d^	7.5	10.9
Tribenuron-methyl	22.5	0	0	0	0	0	0	0	0	6199 (36)^ab^	6054 (39)^cd^	10.9	14.1
Hand-weeding	—	—	—	—	—	—	—	—	—	6734 (214)^ab^	6488 (84)^ab^	20.5	22.3
Weedy control	—	—	—	—	—	—	—	—	—	5590 (250)^c^	5305 (90)^e^	—	—

^a^Visual estimates for crop injury were performed at 3, 5, 15, and 30 DAT, using a 0% (no crop injury) and 100% (plant death) scale. ^b^Different significance between the wheat injuries or wheat yields of 2 years according to Fisher’s protected LSD test at the 0.05 level. *Significant; NS, not significant. ^c^The following different letters represent different significance at the P < 0.05 level according to Fisher’s protected LSD test.

## Discussion

The chemical structure of the herbicide QYM201 (Fig. 1), is similar to that of topramezone, which is a typical HPPD-inhibitor^22^. Furthermore, weeds or crops that have been treated with QYM201 present with typical HPPD inhibitor injury characteristics. Thus QYM201 is suggested to be a novel member of the group of chemicals that inhibits 4-hydroxyphenylpyruvate dioxygenase^15^.

The greenhouse bioassay results indicated that the weed control spectrum of QYM201 was broader than that of most commonly used herbicides in wheat fields; at all rates of application, QYM201 was able to control both grass weeds and broadleaf weeds. Post emergence applications of herbicides such as fluroxypyr and tribenuron-methyl are highly efficient against a large number of broadleaf weeds but provide only limited control for grass weeds^23,24^. Whereas fenoxaprop-P-ethyl and mesosulfuron-methyl are sufficiently effective against many grass weeds, they are not effective for broadleaf weeds^25^. The ability of QYM201 to control both grass and broadleaf weeds may make it a preferred choice over any other ordinary herbicides for weed control in wheat fields. Importantly, we found that QYM201 was highly effective in its control of *A. aequalis* and *A. japonicus*, which are the most harmful weeds to wheat yield worldwide. The widespread application of herbicides has led to the rapid evolution of *A. aequalis* and *A. japonicus* herbicide tolerance throughout the world. In some areas of China, *A. aequalis* has developed resistance to ALS inhibitors^26,27^, and/or ACCase inhibitors^27,28^, while *A. japonicus* has developed resistance to chlorsulfuron^28,29^, to fenoxaprop-P-ethyl^30–32^, to isoproturon^30^, and/or to pinoxaden^31^. Therefore, QYM201 will be helpful in controlling these resistant weeds. However, more attention should be given to preventing the development of resistance to QYM201, especially in *A. aequalis* or *A. japonicus*, by alternately using herbicides at different sites of action, promoting the diversity of crop cultivation, using biological controls, and the rational mixing of herbicides^32^. However, the efficacy of QYM201 on a greater number of weed species that occur in wheat fields needs to be tested before recommendation of its widespread application.

In crop safety experiments, under all tested application rates, QYM201 was safe for most of the 17 tested hybrid wheat varieties. These results strongly suggested that QYM201 is an excellent alternative herbicide for controlling weeds in wheat fields. Moreover, the SI values were identified for JM22, LX66, TN18, and 4 common weeds that occur in wheat fields. It is well known that herbicides are more selective between crops and weeds when the SI value is greater than 1.0^33^, and herbicides can be safely used in crops when the SI value increases over 2.0^34^. In this study, we found that QYM201 was safe for JM22, LX66, and TN18 against *A. aequalis*, *A. japonicus*, *C. bursa-pastoris*, and *M. aquaticum* when POST was applied, with SI values from 5.7 to 16.6. However, the safety of QYM201 for use on other wheat hybrids should be assessed in further experiments in view of the complex distribution of wheat hybrids throughout different areas of China.

The 2-year field experiments demonstrated that the herbicide QYM201 had good efficacy against *A. aequalis, D. sophia*, and *M. aquaticum* with POST at doses of 90–270 g a.i. ha^−1^. Previous field studies further indicate that QYM201 has potential as a POST for weed control. Cheng *et al*.^35^ report that weeds die more slowly than in the glasshouse, which was in accordance with our research. This might be owing to greater weed leaf-age and lower temperatures in the field. Furthermore, no obvious damage to wheat plants was observed during the 2 experimental years in any QYM201 treatments. Moreover, the effect of QYM201 on crop yield was characterized; results showed that wheat yields were higher in 2016 than in 2017 (Table 5). The differences between the data received might be owing to the lower weed density occurring in the experimental sites in 2016. Other factors such as different environmental conditions could also have caused these differences. Wheat yield was increased for all the QYM201 treatments; furthermore, the wheat yield at 270 g a.i. ha^−1^ was not much different from that at 180 g a.i. ha^−1^. According to our research, all of the facts indicate that the recommended dosage of QYM201 is 90 to 180 g a.i.ha^−1^. Field results indicated that the hand-weeding plots had the highest yield among all the treatments; however, the cost of labor make this economically unattractive^36,37^. It is commonly agreed that the combination of chemical measures with other agronomic methods may result in economical and effective control of weeds in wheat fields.

In summary, results from greenhouse and field studies indicated that QYM201 has good potential as an efficient broad-spectrum herbicide for controlling weeds in wheat fields. Under the challenge of controlling multiple herbicide resistance in weeds, the novel structure of this herbicide and its different mode of action could be an ideal option for weed control, especially for resistant weed species in wheat fields.

## Methods

### Herbicide used

QYM201 (Kingagroot, Qingdao, China) with 98% purity and 6% oil dispersion (OD) was provided by Qingdao Kingagroot Chemicals Co., Ltd. Control herbicides fenoxapro-P-ethyl 69 g L^−1^ and tribenuron-methyl 75% WDG were provided by Bayer Crop Science Co., Ltd. and Jiangsu Rotam Chemicals Co., Ltd., respectively. To obtain a series of concentrations, QYM201 98% technical material (TC) was dissolved in ethanol and diluted with 0.1% Tween-80 solutions. QYM201 6% OD and the 2 control herbicides were dissolved and diluted with deionized water.

### Greenhouse experiment

Weed seeds of *A. aequalis*, *A. japonicus, Veronica didyma* Tenore, *D. Sophia*, and *Becmannia syzigachne* (Steud.) Fern were collected from Jiangsu province and the other 13 weed species were collected from Henan province, China, in 2014 (Table 1). All weed species seed germination rates were >85%. All wheat hybrids used in this study can be found in the agricultural seed market and they are listed in Table 2. Germination rates of all wheat seeds were >80%. All greenhouse conditions involved were similar to those in a previous experiment^38^. Experiments were executed at Shandong Agricultural University, Tai’an, China. Weed and wheat seeds were immersed in a petri dish containing distilled water and placed in a 12 h photoperiod and 20 °C growth chamber (Model RXZ, Ningbojiangnan Instrument Factory, Ningbo, China) to accelerate germination before planting. After visualization of seed radicles, 15–30 seeds were sown below the soil surface per plastic pot (160 mm diameter and 130 mm height). After weed emergence, the seedlings were thinned to 10 plants per plastic pot. At the 3–5 leaf stage the seedlings were treated with QYM201 using an auto spraying tower (Model ASS-4, National Agricultural Information Engineering and Technology Center of China) at a spray pressure of 0.275 MPa with 450 L ha^−1^ spray volume. All greenhouse experiments had replications and were repeated once.

### Effectiveness of weed control

All 18 tested weed species were treated with QYM201 at dosage rates of 90 and 135 g a.i. ha^−1^, and an untreated control was designed for each weed species. After 28 days of treatment, the surviving weeds were cut off at the soil surface and placed in a labeled paper bag, put in an oven at 80 °C for 72 h, and finally the dry weights were recorded^39^. Other experimental conditions were consistent with those described above for the greenhouse experiment.

### Wheat hybrid tolerance

All wheat hybrids were treated with QYM201 at 360 and 540 g a.i. ha^−1^, and a non-treated control was also designed. After 28 days of treatment, wheat plants were cut off and put in an oven at 80 °C for 72 h, and then dry weights were recorded. In addition, the degree of herbicide damage to wheat seedlings was also recorded and expressed as values from 0 to 100%: 0% indicated no damage and 100% indicated total death^31^. Other experimental conditions were consistent with those described above for the greenhouse experiment.

### Selectivity index (SI)

The selectivity index refers to the ratio between the concentrations that caused 10% growth inhibition of crops and 90% growth inhibition in weeds^40^. Three commonly cultivated wheat hybrids [Jimai 22 (JM 22), Liangxing 66 (LX 66), and Tainong 18 (TN 18)] in China and 4 widespread weeds (*A. aequalis*, *A. japonicas*, *C. bursa-pastoris*, and *M. aquaticum*) that occur in wheat fields were selected for testing. In order to obtain the SI values between wheat and weed species under QYM201 application, JM 22, LX 66, and TN 18 were treated at rates of 0, 270, 405, 607, 911, 1366, 2050, and 3075 g a.i. ha^−1^; *A. aequalis* and *M. aquaticum* were treated with doses of 0, 15, 30, 45, 60, and 75 g a.i. ha^−1^; *A. japonicus* was sprayed at concentrations of 0, 45, 60, 75, 90, and 135 g a.i. ha^−1^; and *C. bursa-pastoris* was treated at rates of 0, 15, 60, 90, and 135 g a.i. ha^−1^. These experiments were carried out simultaneously under the same experimental conditions. After 28 days of treatment, to record plant dry weights, shoots were harvested and put in an oven at 80 °C for 72 h. Other conditions during the experiments were consistent with those described above.

### Field experiment

Field experiments were conducted in 2016 and 2017 at Ningyang, Tai’an which is situated in the northern winter wheat growing areas. The soil type was loam with 1.79% organic matter, pH 7.5, and a widely grown wheat hybrid Tainong 18 was tested in this study. On October 2, 2015, and October 9, 2016, winter wheat was mechanically sown in rows at 15 cm intervals at a seeding rate of 225 kg ha^−1^. The weed species that were common in this area during the 2 experimental years were *A. aequalis, D. Sophia, and M. aquaticum*. The average densities for *A. aequalis* were 20 and 31 plants per m^2^, 18 and 28 plants per m^2^ for *D. sophia*, and 12 and 19 plants per m^2^ for *M. aquaticum*, respectively, in 2016 and 2017. Before wheat sowing, diammonium phosphate was applied at a ratio of 450 kg ha^−1^, and on March 3, 2016, and March 15, 2017 urea fertilizer was applied at a ratio of 375 kg ha^−1^ at the wheat turning green stage. The monthly temperatures and precipitation at the site during the experimental period are shown in Table 6.

**Table 6:** Monthly air temperatures and total precipitation at Tai’an experimental site in Shandong, China, during the period from QYM201 application to wheat harvest in 2016 and 2017.

Month	Air temperature (°C)						Total precipitation (mm)	
	Maximum		Minimum		Mean			
	2016	2017	2016	2017	2016	2017	2016	2017
March	16.7	14.7	3.2	2.3	10.2	8.5	0	15.0
April	24.1	22.5	12.2	9.5	17.7	16.0	6.9	33.3
May	35.9	29.7	14.9	16.5	20.3	23.0	48.7	24.8
June	30.7	30.6	20.2	19.6	25.1	24.9	167.8	62.4

All treatments were arranged in a randomized complete block design and repeated 4 times. The area of each plot was 20 m^2^ (4 m wide and 5 m long). This experiment contained a total of 8 treatments and there were 4 application rates of QYM201 (90, 135, 180, and 270 g a.i. ha^−1^); a single concentration of fenoxapro-P-ethyl (at a rate of 50 g a.i. ha^−1^) and tribenuron-methyl (at a dose of 25 g a.i. ha^−1^), respectively; a hand-weeded control (using hand hoes at 0, 15, 30 and 45 DAT) and an untreated control (Table 4). On March 21, 2016, and March 16, 2017, weeds were sprayed with herbicides at the 7 to 8 leaf stage. The average temperatures on the days of application were 13.2 °C and 9.7 °C, respectively. Herbicides were applied using a backpack sprayer (Bellspray Inc., Opelousa, LA) fitted with a single 8002 VS nozzle (Teejet Technologies, Wheaton, IL) in 450 L ha^−1^ of water.

Visual estimates for crop injury were performed at 3, 5, 15, and 30 DAT, using a 0% (no crop injury) and 100% (plant death) scale. Visual estimates for weed control were recorded after 45 days of treatment, using a 0% (no weed control) to 100% (complete weed control) scale^30^. In each test plot a random sample area of 0.33 m^2^ was surveyed at 3 sample points. The number of healthy plants of 3 weed species at each sample point was investigated at 0, 15, 30, and 45 DAT, and the fresh weight of weeds was recorded while investigating the number of weeds at 45 DAT. At the time of wheat harvesting, 3 samples were taken per plot and weighed to evaluate the grain yield of each plot; the resulting wheat yield was expressed as kg/ha.

### Statistical analysis

All greenhouse experiment data were subjected to Analysis of Variance (hereafter referred to as ANOVA; Version 22.0; IBM Corporation, Armonk, NY). Data were pooled because there was no significant (P > 0.05) interaction with the 2 replicate treatments, and means were separated using Fisher’s protected LSD tests at the 0.05 level. All regression analyses were performed using SigmaPlot software (Version 13.0; Systat Software Inc., CA, USA). To evaluate weed control and assess the dose of QYM201 required for 90% weed control, regression of weed dry matter over herbicide dose was performed using the 4 parameter log-logistic model described by Seefeldt *et al*.^41^:

1 $$y\,=\,c\,+\,(d\,-\,c)/\{1\,+\,\exp [b(\mathrm{log}\,x\,-\,\mathrm{log}\,G{R}_{{50}})]\}$$

where *b* is the slope of the line, *c* is the lower limit, *d* is the upper limit. *x* is the herbicide dose, *GR*_50_ is the dose giving 50% response, and *y* is the growth response (percentage of the untreated control).

*GR*_10_, *GR*_50_, and *GR*_90_ values were calculated according to regression parameters^18^. The SI values of QYM201 were calculated by the following equation:

2 $$S{I}_{(10,90)}\,=\,G{R}_{10(crop)}/G{R}_{90(weed)}$$

where *GR*_10_ is the dose of wheat growth reduction by 10%, and *GR*_90_ is the dose of weeds growth reduction by 90%.

Field experiment data were subjected to ANOVA, and means were separated using Fisher’s protected LSD tests at the 0.05 level. Treatment interactions of the 2 years were not significant (P > 0.05), therefore the data were pooled by the year.

## Data Availability

All data generated or analyzed in this study are included in the Supplementary Information files.
